# Application of 5-aminolevulinic acid-mediated Waterlase-assisted photodynamic therapy in the treatment of oral leukoplakia

**DOI:** 10.1038/s41598-022-13497-3

**Published:** 2022-06-07

**Authors:** Jiali Ou, Yijun Gao, Huan Li, Tianyou Ling, Xiaoyan Xie

**Affiliations:** Department of Stomatology, The Second Xiangya Hospital, Central South University, 139 Renmin Middle Road, Changsha, 410011 People’s Republic of China

**Keywords:** Diseases, Medical research, Risk factors, Signs and symptoms

## Abstract

Photodynamic therapy (PDT) is an alternative microinvasive approach with satisfying results in the treatment of oral leukoplakia (OL). PDT combined with laser irradiation shows promise, safety and efficacy in treating OL. The efficacy of waterlase (YSGG) combined with PDT was studied by brush and tissue biopsy. Seventy-one patients with histologically diagnosed OL were enrolled, including patients with mild to moderate dysplasia, severe dysplasia and various dysplastic tissues. Patients were evaluated at baseline (t0), the end of treatment (t1) and 1 year later (t2). At t1, PDT showed a significant therapeutic effect on OL with mild to moderate dysplasia. Clinical and histological examinations revealed 60 cases (84.51%) of complete remission and 11 cases (15.49%) of partial remission. On brush biopsy, all PDT-treated patients showed reduced aneuploidy and normal histological findings. Unfortunately, at t2, 9 patients relapsed with OL, which may be related to continued smoking and betel nut chewing. At t2, 5 patients developed new severe epithelial dysplasia and even carcinoma in situ in other areas, mostly the tongue. ALA-mediated PDT combined with YSGG is effective in treating OL, particularly that with mild to moderate dysplasia. However, severe dysplasia may present undesirable effects, and the mechanism remains to be further investigated. ALA-mediated PDT combined with YSGG provides a new method for OL treatment.

## Introduction

Oral leukoplakia (OL), according to the World Health Organization (WHO), is defined as a mainly white patch, plaque, or lesion on the oral mucosa that cannot be certainly classified clinically or histologically as any other ascertainable disorder^1–4^. OL is a well-known oral potentially malignant disorder (OPMD) with a potential risk of malignancy. It has been reported that 15.8–48.0% of patients with oral squamous cell carcinoma (OSCC) have a history of OL when diagnosed^5^. According to a systematic review of 24 retrospective studies, the prevalence rate of OL is 0.13–34%^6^.

The main goal of the treatment of OL is to prevent malignant transformation and even reduce or eliminate lesions^7^. There are probably two traditional methods to treat OL, namely, conservative treatment and surgical resection. Medical interventions, such as vitamin A, retinoids, beta-carotene or bleomycin cannot reduce the possibility of oral cancer development^8^ and have low efficiency with a long treatment time^1,9^. Therefore, surgical methods, including conventional excision, cryosurgery therapy, electrocauterization and various forms of laser ablation, are the preferred choice in OL management for many clinicians^8^.

However, when the diseased area extends to multiple locations or is located in potentially important functional areas, surgical methods may be impractical, as they can cause large tissue defects, therefore influencing function and aesthetics^7^. Additionally, data have shown that OL has a 13–42% recurrence rate after surgical removal^1,9,10^. Photodynamic therapy (PDT) is a less invasive method that can successfully be applied in the treatment of premalignant and malignant disorders of the skin, digestive tract, lungs, and genitourinary mucosa with vastly reduced morbidity and disfigurement. Since PDT is a cold photochemical process, there is no tissue heating and a much lower risk of damaging the integrity of the underlying functional structures compared to thermal laser techniques and other invasive approaches. PDT is preferred by patients due to its good effect, less postoperative pain, slight edema, and few areas of scarring^11–13^ and the fact that it can be repeated indefinitely at the same site without cumulative toxicity.

The technique of PDT is simple and consists of three fundamental elements, including oxygen, a photosensitizer and visible light at a specific wavelength. Aminolevulinic acid (ALA) serves as an endogenous precursor of the photosensitizer protoporphyrin IX (PpIX), shows superb tissue selectivity and targets proliferative epithelial cells and is thus used for the treatment of oral premalignant and malignant lesions^7^. Owing to the poor penetration of ALA, the pretreatment of lesions, for example, scaling with a scalpel and vaporization with a CO_2_ laser which creates microscopic vertical holes serving as channels with connections between treatment zones and upper tissues^14^, is usually performed before delivering topical PDT^1,15^. Waterlase (YSGG) has the feature of “point clearance” and generates vertical channels to provide direct delivery and better distribution and hence can also be used to pretreat OPMDs to enhance ALA penetration^15^. YSGG pretreatment has some advantages, such as decreasing bleeding; reducing pain, edema and risk of infection; and accelerating healing^16^.

In our study, 71 patients with OL were treated with YSGG-PDT, with evaluation of the effects at baseline (t0), the end of treatment (t1) and 1 year later (t2). Our investigation will provide a reference for the application of YSGG-PDT in the treatment of OL.

## Materials and methods

### Sample collection

This study was approved by the Institutional Ethics Committee of the Second Xiangya Hospital of Central South University of China (20200606). All participants understood the nature of this study and provided written informed consent. Photographs of each participant’s lesions were carefully obtained, and each participant underwent clinical examination, brush biopsy and tissue biopsy of the lesion site.

Seventy-one patients underwent treatment at the Department of Stomatology Center of the Second Xiangya Hospital, Central South University of China. All patients with clinically and histologically diagnosed OL were enrolled in this study. Eligible OL patients were recruited from the Department of Stomatology of the Second Xiangya Hospital, Central South University of China between July 2019 and June 2021. The age of the patients was between 18 and 75 years. All patients had untreated lesions identified on biopsy as mild, moderate or severe epithelial dysplasia as well as histologically verified OL.

### Exclusion criteria

The exclusion criteria were as follows: age under 18 years or beyond 75 years; allergy to photosensitizers; breast feeding or pregnancy; other oral mucosal diseases; oral cancer, ranging from microinvasive cancer to invasive cancer to metastatic cancer, etc.; coagulopathy; any psychosis; uncontrolled severe systemic diseases (such as uncontrolled diabetes, hypertension, heart disease, severe kidney and liver damage, or malignant tumors); and inability to understand or tolerate treatment.

### Treatment protocol

Patients were evaluated at baseline, the end of treatment (t1) and 1 year later (t2). All treated lesions were recorded and photographed at each visit. Brush biopsy was utilized to identify patients with mild, moderate or severe epithelial dysplasia. The brush head was placed tangentially to the lesion site and rotated at least eight to ten times. Subsequently, the brush was bent, and the head was washed out fully in the preservation vial. The collection of cells was then sent for DNA aneuploid analysis. Then, an incisional biopsy was performed to verify the absence of cancerization. The histopathological outcomes showed “hyperkeratosis and dyskeratosis, no detectable cancerization” in all examined lesions, confirming a clinical diagnosis of OL.

Without local anesthesia, YSGG (Waterlase Biolase Technology) (the wavelength was 2780 nm, the output power ranged between 1.0 and 2.0 w; pulse repetition rate was 20–35 Hz in pulsed mode, and the water/air proportion was set at 20%/40%) was applied to create exposed vertical channels to accelerate photosensitizer penetration and absorption in noncontact mode. 5-Aminolevulinic acid (ALA, Zhangjiang Pharmaceuticals, Shanghai, China) was dissolved with temperature-sensitive gel to generate a 20% gel immediately before use. Prior to PDT, a thin cotton swab soaked with the prepared photosensitizer precursor gel was gently placed over the lesion with a margin of approximately 2–3 mm from the surrounding normal area for 3 h after salivary isolation was established. Additionally, a starch film was layered above the cotton swab to facilitate the adhesion of the photosensitizer precursor to the oral mucosa. Furthermore, food grade clingfilm and thick gauze were used to cover the targeted area and to protect the photosensitizer from dilution by neither blood nor other exudates. Finally, suction pipe fixed by the bite block continued working in the opposite posterior teeth area while keeping the head toward the bite block to avoid contamination by saliva.

After 3 h, illumination was performed with a red light (LED-IB photodynamic therapy instrument, Yage Optic and Electronic Technique Co., Ltd., Wuhan, China) at a wavelength of 635 nm. The output power was 120 J/cm^2^, and 80 mW/cm^2^ fluence was applied for approximately 25 min/illuminated spot. The fiber optic ended at a microlens that focused the light radiation equally and homogeneously onto a spot 2 cm in diameter. Each spot overlapped with adjacent spots by 3–5 mm to ensure complete coverage of the lesion. To maximize the therapeutic effect under patient tolerance, the lesion was artificially divided into multiple treatment areas. The whole area covered in each treatment stage was confined to a diameter of 2–3 cm. Patients were evaluated at baseline (t0), the end of treatment (t1), and 1 year from the end of treatment (t2). All treated zones were photographed to evaluate (by both brush and tissue biopsy) the lesion size and its change during the treatment period. Care was taken not to stretch or distort the mucosa, potentially altering the lesion size.

### Thin-layer liquid-based cytology test (TCT)

We used a liquid-based thin-layer cell detection system to detect oral cells and perform cytological classification and diagnosis. TCT was used to determine cell types, pathogens, etc. Atypical squamous cells, low-grade squamous intraepithelial lesions, and OSCC were considered to indicate a positive diagnosis.

### DNA ploidy analysis

DNA ploidy was analyzed by using the LD DNA-ICM system (Landing, Wuhan). The average cell DNA content in each sample compared with that of normal cells was defined as the DNA index (DI). Lesions with DI < 1.1 were considered diploid, and lesions with DI > 1.2 were defined as aneuploid.

### Hematoxylin–eosin (HE) staining

First, paraffin sections were deparaffinized. Then, the sections were stained in hematoxylin solution for 3–8 min and washed with running water. The sections were then stained with eosin solution for 1–3 min. Finally, the sections were sequentially placed into different concentrations of alcohol for dehydration, sealed with neutral gum and observed under a microscope.

### Statistical analysis

R software was used for statistical analysis. The zones of the lesions (mm^2^) at t0, t1 and t2 were compared by the Mann–Whitney U test. When p < 0.05, the difference was considered significant. The RECIST criteria were used to analyze the images and assess the efficacy of the treatment as follows: complete remission (CR) is defined as the absence of detectable lesions confirmed by clinical evaluation (including both brush and tissue biopsy) and 100% reduction of the lesion area on computerized analysis; partial remission (PR) is defined as at least a 20% reduction of the lesion area.

### Ethics approval

Ethics Approval was given by the Medical Ethics Committee of Second Xiangya Hospital, Central South University, 2020606. We confirmed that all methods were performed in accordance with the relevant guidelines and regulations.

### Informed consent

Informed consent was obtained from all individual participants included in the study.

## Results

### Basic patient characteristics

As shown in Table 1, we collected clinical samples from 71 patients, including 69 males and 2 females. Seventy-one patients were “heavy lifelong smokers”, 37 were “heavy lifelong drinkers” and 20 were “heavy lifelong areca nut chewers”. Primary lesions were mainly observed in the tongue (n = 47), floor of the mouth (n = 6) and buccal areas (n = 18). At t1, 10 patients still smoked, 15 patients still drank, and 1 patient chewed areca nuts.

**Table 1:** Patients' clinical characteristics.

Characteristical	Number of each grade of dysplasia			
	Mild	Moderate	Severe	Amount
**Gender**				
Male	29	31	9	69
Female	0	2	0	2
Heavy lifelong smokers	29	33	9	71
Heavy lifelong drinkers	8	20	9	37
Heavy lifelong chewing areca nut	1	10	9	20
**Location**				
Tongue	24	18	5	47
Buccal	2	12	4	18
Mouth	3	3	0	6
**t1**				
Still smoking	0	5	5	10
Still drinking	0	11	4	15
Still chewing areca nut	0	0	1	1

### Effect of YSGG-PDT on OL

**Figure 1:**
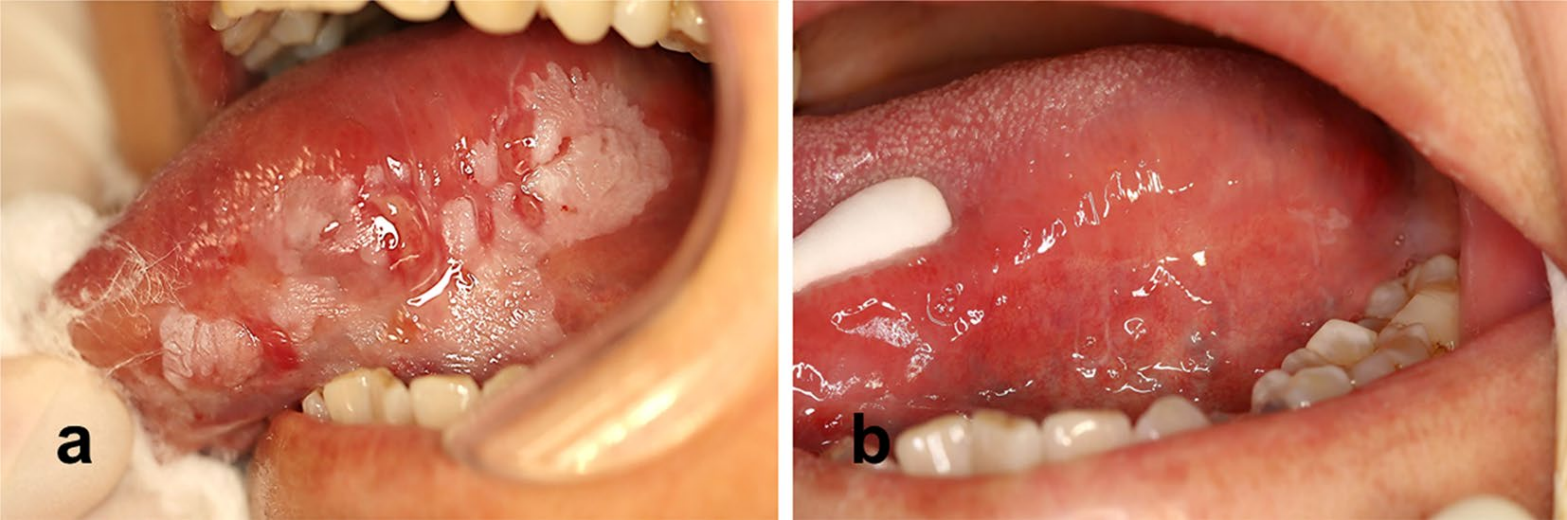
Preoperative (**a**) and postoperative (**b**) treatment of OL patients. The patient’s oral lesions were recorded by taking pictures.

We performed YSGG-PDT on OL patients and found that YSGG-PDT was 100% effective in treating dysplastic OL. Figure 1 shows photographs taken before and after the treatment of OL patients. The leukoplakia of OL patients treated with YSGG-PDT basically disappeared. We clinically treated 71 patients and found that 51 patients with mild to moderate epithelial dysplasia achieved complete remission (CR), while 11 patients achieved partial remission (PR). The primary lesions of 9 patients with severe epithelial dysplasia all showed CR (Table 2). These results suggest that YSGG-PDT has a CR rate of 84.51% for all OL patients.

**Table 2:** The therapeutic effect of YSGG-PDT on OL.

Results of treatment	Number of people
Complete remission (CR)	60 (84.51%)
Partial remission (PR)	11 (15.49%)

### Effect of YSGG-PDT on DNA ploidy in OL patients

**Figure 2:**
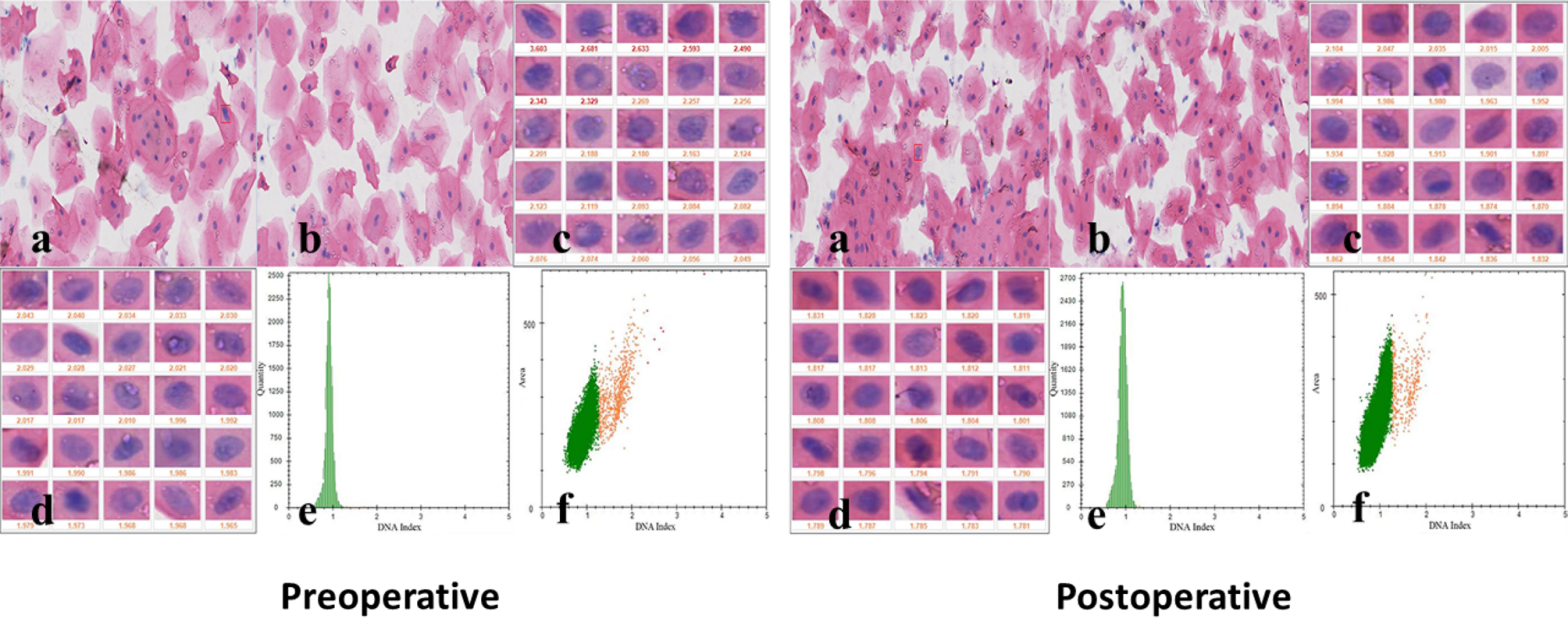
DNA aneuploid analysis of patients of preoperative and postoperative treatment. The oral cells before and after treatment of YSGG-PDT were analyzed by TCT, and cytological classification and diagnosis were performed. DNA ploidy was used to analyze the changes in the number of DNA ploidy in oral cells before and after YSGG-PDT treatment. (**a**) The staining quality of cell smear preparation (Staining Feulgen, lens 100×); (**b**) The morphology of normal cells (Staining Feulgen, lens 100×); (**c**) The morphology of abnormal oral cells (Staining Feulgen, lens 400×); (**d**) Other abnormal cells, including pathogenic microorganisms (fungi, trichomonas, parasites, etc.) (Staining Feulgen, lens 400×); (**e**) The histogram shows the distribution of cells of different volumes and abnormal cell peaks (double peaks, three peaks, multiple peaks, abnormal peaks, etc.); (**f**) The scatter diagram shows the number of cells of different DNA ploidy.

**Figure 3:**
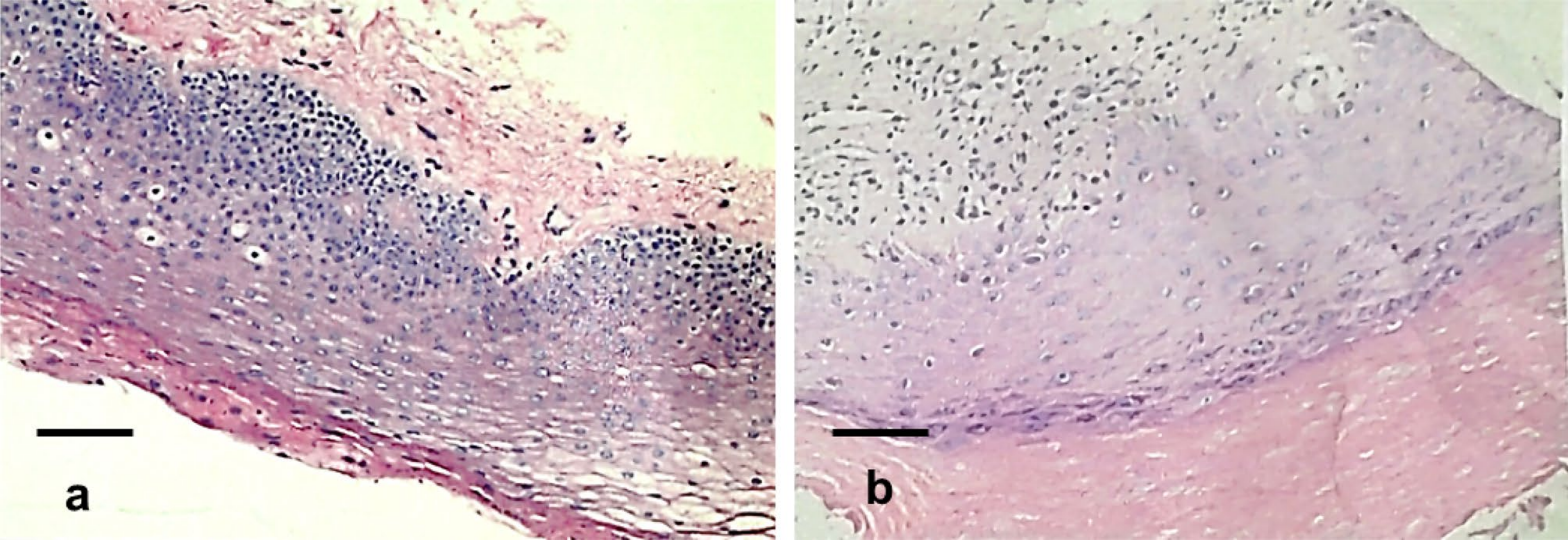
HE stains of patients of preoperative (**a**) and postoperative (**b**) treatment. The pathological changes of oral tissue before and after YSGG-PDT treatment were analyzed by HE staining (scale bar: left panel, 50 μm; right panel, 125 μm. Magnification: left panel, HE × 40; right panel, HE × 100).

We further examined the oral tissue cytology of the patients before and after treatment. The TCT test showed that there were no nuclear heterogeneous cells among the oral cavity cells of the patients before or after treatment, but there were a small number of inflammatory cells (Fig. 2). DNA ploidy analysis showed reduced aneuploidy after YSGG-PDT treatment in all patients (Fig. 2). At the same time, HE staining examination showed that the in situ epithelial dysplasia of the patients' oral tissue disappeared after YSGG-PDT (Fig. 3). These results indicate that YSGG-PDT has a good effect in OL patients.

### Effect of YSGG-PDT on follow-up

**Figure 4:**
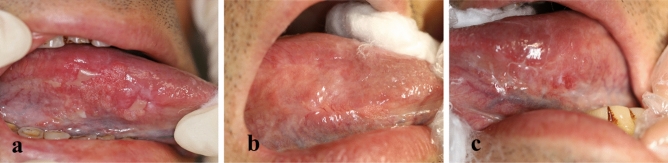
OL patients with severe dysplasia at t0 (**a**), t1 (**b**) and t2 (**c**), New lesions occured in new areas). The lesion changes at t0, t1, and t2 in YSGG-PDT treatment of OL patients with severe dysplasia were recorded by taking pictures.

**Figure 5:**
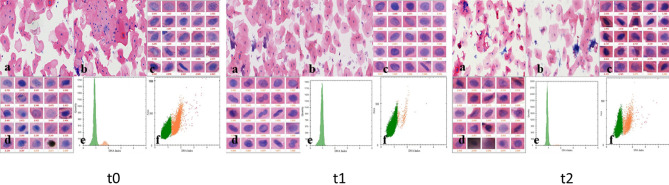
DNA aneuploid analysis of OL patients with severe dysplasia at t0, t1 and t2 (DNA aneuploid analysis in new areas). The cytological classification and diagnosis of YSGG-PDT in patients with severe dysplasia at t0, t1, and t2 were analyzed by TCT. DNA ploidy was used to analyze the changes of cell DNA ploidy at t0, t1, and t2 in YSGG-PDT treatment of OL patients with severe dysplasia. (**a** The staining quality of cell smear preparation (Staining Feulgen, lens  ×100); **b** the morphology of normal cells (Staining Feulgen, lens  ×100); **c** the morphology of abnormal oral cells (Staining Feulgen, lens  ×400); **d** other abnormal cells, including pathogenic microorganisms (fungi, trichomonas, parasites, etc.) (Staining Feulgen, lens  ×400); **e** the histogram shows the distribution of cells of different volumes and abnormal cell peaks (double peaks, three peaks, multiple peaks, abnormal peaks, etc.); **f** the scatter diagram shows the number of cells of different DNA ploidy.)

**Figure 6:**
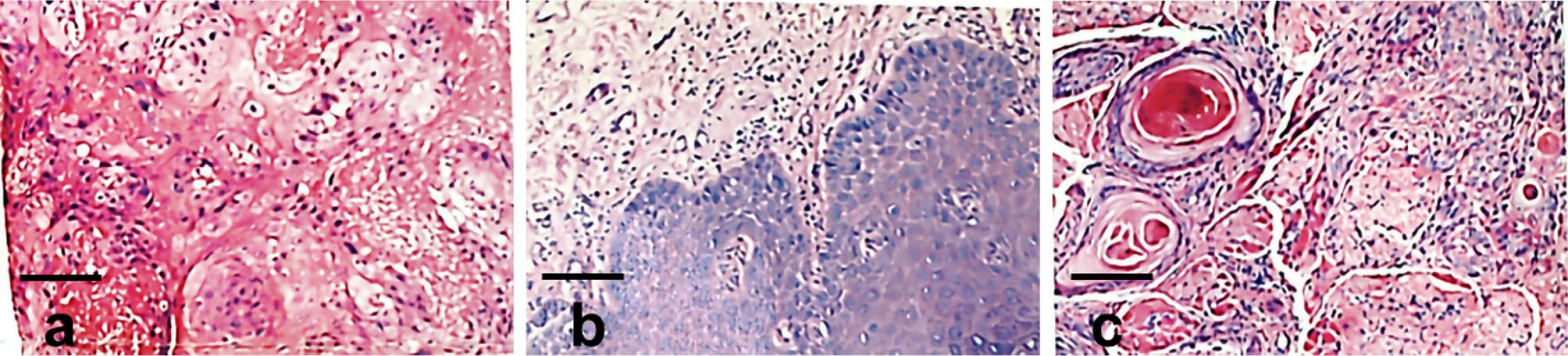
HE stains of OL patients with severe dysplasia at t0 (**a**), t1 (**b**) and t2 (**c** HE stains in new areas). The pathological characteristics of the oral lesions at t0, t1, and t2 in the YSGG-PDT treatment of OL patients with severe dysplasia were analyzed by HE staining. Scale bar 500 μm. Magnification: HE × 400.

**Figure 7:**
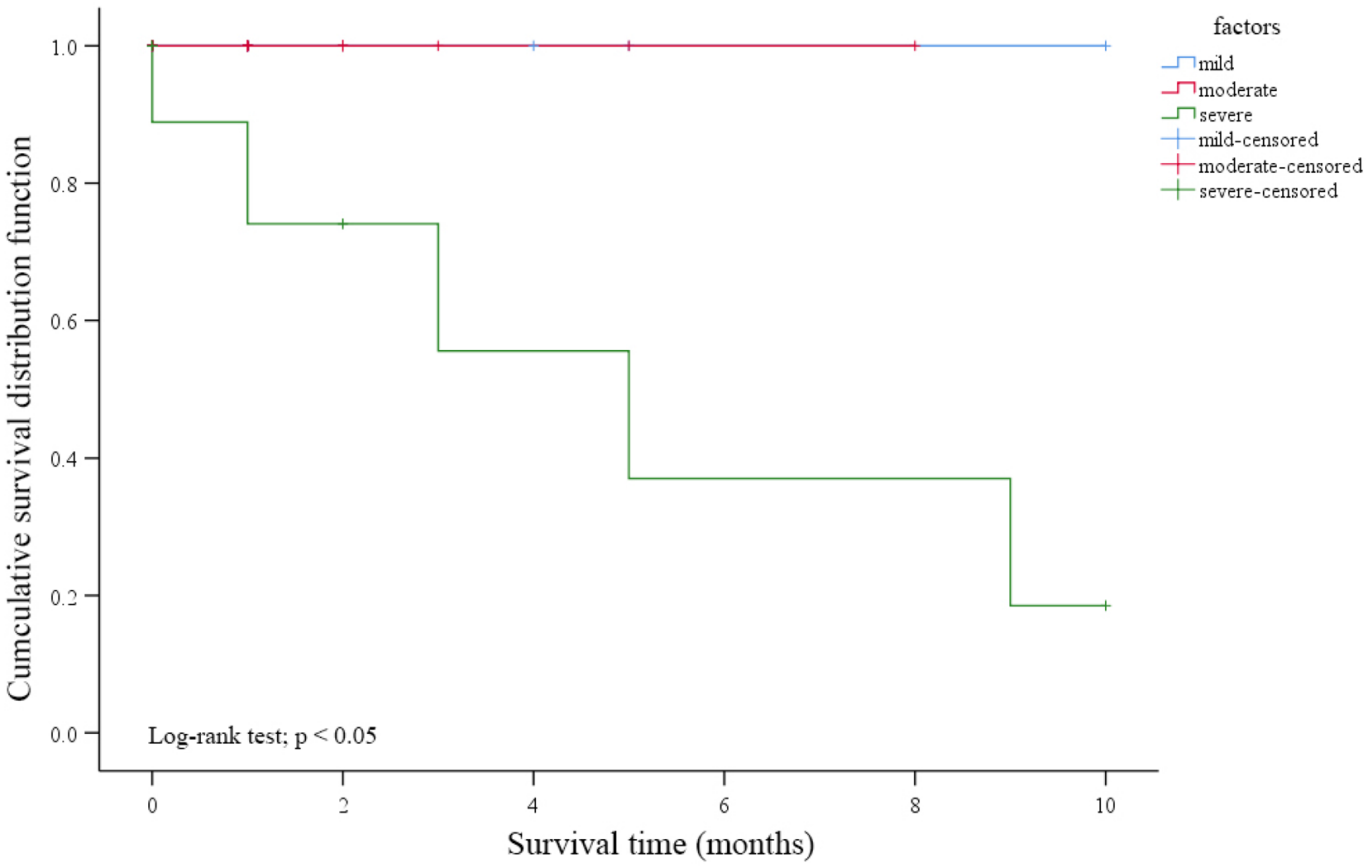
Kaplan–Meier survival curves for each grade of dysplasia after PDT treatment. Severe epithelial dysplasia patients were a significantly higher risk of developing new severe epithelial dysplasia and even carcinoma.

Unfortunately, after continuing patient follow-up, at t2, 4 OL patients with mild to moderate dysplasia showed recurrence (Table 3). We speculate that this may be related to continued smoking, drinking, and betel nut chewing after treatment. At the same time, 5 patients with OL and severe dysplasia developed new lesions, such as ulcers (Fig. 4). TCT analysis of these 5 patients revealed that no nuclear heterogeneous cells were seen at t1, while atypical squamous epithelial cells were observed at t2 (Fig. 5). DNA ploidy analysis showed that compared with t0, the number of aneuploidies was significantly reduced at t1, while the number of aneuploidies was obviously increased at t2 (Fig. 5). HE staining confirmed that at t2, severe epithelial dysplasia and even carcinoma in situ appeared in other parts of the oral cavity (Fig. 6), mostly on the tongue. The Kaplan–Meier curves showed the long-term effect of YSGG-PDT (Fig. 7).

**Table 3:** Follow-up effects of YSGG-PDT treatment.

Detection	t0	t1		t2			
Number	71	60	11	60		11	
				5	51	7	4
TCT	Atypical squamous epithelial cells	No nuclear heterogeneous cells	No nuclear heterogeneous cells	Atypical squamous epithelial cells	No nuclear heterogeneous cells		No nuclear heterogeneous cells
Epithelial dysplasia	Yes	No	No	Yes	No		Yes
Lesion	Oral leukoplakia	Disappeared	Oral leukoplakia	Epithelial dysplasia and carcinoma	Disappeared		Oral leukoplakia

## Discussion

### YSGG pretreatment combined with PDT

ALA-PDT is currently a notable treatment selection for oral premalignant lesions because it results in good functional and cosmetic outcomes with the lowest scarring, slight postoperative pain and mild edema. However, ALA has been reported to have poor penetration, which causes a reduced effect of PDT^15^. A previous study showed a CR rate of only 33.33% when using PDT with topically administered ALA to treat OL patients^1^. Therefore, pretreatment is clinically required to promote PDT efficacy by producing an alternative pathway to accelerate the absorption of photosensitizer precursors.

Currently, CO_2_ laser is usually used to pretreat malignant lesions for vaporization with the removal of stratum corneum infractions and partitions^17^. The reported CR to ALA-PDT combined with CO_2_ laser was 78.3%, and the PR rate was 21.7%. The recurrence rate was 17.4% over a follow-up period of 12 months^15^. However, the CO_2_ laser can cause thermal side effects^18^ and induce dysplastic-like epithelial artifacts^19^. In the study, we applied YSGG pretreatment and found a response of 84.51% (60/71) CR and 15.49% (11/71) PR and recurrence of 12.68%. These results demonstrate that YSGG-PDT is more effective than CO_2_ laser-PDT. Moreover, the pain and local ulcer observed in the study are also notably minimized and rapidly recovered compared with the use of other lasers, probably for the energy from YSGG is absorbed effectively by tissue and water, with minimal tissue charring and collateral thermal damage.

In summary, our study shows that combining PDT with YSGG is effective in the treatment of OL, which is a promising treatment method for achieving excellent outcomes for patients.

### Brush biopsy and DNA aneuploidy detection

A considerable number of OSCC lesions can evolve from precancerous lesions, such as OL and oral submucosal fibrosis (OSF) lesions. Therefore, early identification of high-risk OPMDs and interventions in the precancerous stage related to OSCC can reduce the morbidity and mortality of OSCC and save treatment costs^20^. The current histological evaluation standard is an important method for confirming the diagnosis, and there are no auxiliary tests to replace it. However, biopsy is an aggressive technique with certain surgical implications and high technical requirements for professionals; this process may also have psychological effects on the majority of patients^21^. In contrast, cytology shows promise. Brush biopsy examination is simple and fast^21–23^. Moreover, the results are similar to those of histological examination. Many studies have demonstrated that an increased nuclear size and a reduced cytoplasmic size are helpful indicators of early malignant transformation^24^. Hence, exfoliative cytology is of value for monitoring clinically questionable lesions and for the early detection of malignancy^25–27^.

Furthermore, the mutational spectrum of a malignant tumor changes significantly throughout tumor development^24,27,28^, and approximately 10–80% of cancers display duplication of the whole genome, causing chromosomal alterations^24,26,29–32^. Remmerbach et al. concluded that DNA aneuploidy might reveal histologically obvious malignancy 1–15 months before histology in the clinical setting^33^. Sudbo et al. analyzed previous material and reported that the nuclear DNA content in OL cells may be used to predict the risk of oral epithelial dysplasia up to 5 years before histological diagnosis^23^. Grounded in these findings, they proposed brush biopsy with cytological or DNA cytometric examination for microscopic evaluation of OL or erythroplakia^34^.

In the study, a trend could be observed toward an increase in the number of aneuploidies in patients with severe dysplasia compared with that in patients with mild to moderate dysplasia. The results of DNA aneuploidy analysis agree with the histological outcomes. These findings show that aneuploidy is correlated with increasing severity of dysplasia and predictive of an increased risk of malignant change. Additionally, our results reveal reduced aneuploidy after YSGG-PDT treatment, which suggests a contributory phenomenon regarding a decreasing trend in the connection between YSGG-PDT and the evolution of premalignant or malignant oral lesions. To the best of our knowledge, this is the first study to make use of the efficacy of exfoliative cytology to detect DNA content in ALA-PDT-treated patients with OL. To some extent, our DNA aneuploidy examination indicates that the malignant progression of OL is a characteristic multistep carcinogenic process with the gradual accumulation of DNA aneuploidy. Taking advantage of histological outcomes and the relatively high sensitivity of cytology, the DNA aneuploidy examination of brush biopsies could provide pathognomonic indicators before clinical signs and symptoms appear in early OSCC patients.

### Clear therapeutic effects in patients with mild to moderate epithelial dysplasia

PDT is a cold photochemical reaction. For mild to moderate epithelial dysplastic lesions, PDT exploits photosensitizers that bring forth a photodynamic response to selectively undermine abnormal cells by corresponding light delivery, ultimately resulting in cellular destruction. Developments in photosensitizers and light delivery systems have substantially reduced treatment times and residual photosensitivity while increasing the depth of the effect. Patients feel little pain and are more receptive to such treatments (Supplementary Information).

The present study demonstrates that YSGG-PDT can achieve satisfactory efficacy in treating patients with mild to moderate epithelial dysplasia, with a CR rate of 82.26% and a recurrence rate of 14.52%, which were revealed by both cytological and histological examination. The majority of recurrence cases are attributed to poor compliance of patients who still chew areca nuts and smoke, suggesting that patients with mild to moderate dysplasia who desire more precise and stable treatment need to follow medical advice, for example, quitting smoking, drinking and betel nut chewing.

### Prognosis of patients with severe epithelial dysplasia

Our study shows the significant efficacy, with a CR rate of 100%, of YSGG-PDT at the primary site in patients with OL and severe epithelial dysplasia. However, new lesions with severe epithelial dysplasia or even carcinoma in situ emerged at new sites approximately 1 year after PDT. While we doubt that PDT caused the metastasis of abnormal cells at the original site or induced the malignant transformation of normal cells at the new site, the exact mechanism is still unclear.

Furthermore, we speculated that this outcome might be related to the fact that the laser of the PDT device could not be targeted exclusively at the site of the disease in the mouth; the light could scatter to other sites, resulting in light stimulation. It is also possible that PDT may require different parameter settings for tissues with different degrees of dysplasia to achieve safer and more effective treatment. It is worth noting that most of the new sites of severe epithelial dysplasia or cancer were on the tongue, which may be related to abundant vasculature in the tongue. Of course, the specific mechanism requires further study.

## Conclusion

For patients with mild to moderate dysplasia, YSGG-PDT is effective, with a low recurrence rate. However, for patients with severe epithelial dysplasia, while such treatment has obvious efficacy at the primary site, severe epithelial dysplasia or even primary cancer may occur at other sites. The existing PDT equipment and light devices may need to be improved. Additionally, different parameters may need to be set according to the degree of hyperplasia when using the PDT apparatus.

## Supplementary Information

Supplementary Information.

## Data Availability

All data generated or analysed during this study are included in this published article.
