# Author Correction: Application of 5-aminolevulinic acid-mediated Waterlase-assisted photodynamic therapy in the treatment of oral leukoplakia

**DOI:** 10.1038/s41598-022-26542-y

**Published:** 2022-12-22

**Authors:** Jiali Ou, Yijun Gao, Huan Li, Tianyou Ling, Xiaoyan Xie

**Affiliations:** Department of Stomatology, The Second Xiangya Hospital, Central South University, 139 Renmin Middle Road, Changsha, 410011 People’s Republic of China

Correction to: *Scientific Reports* 10.1038/s41598-022-13497-3, published online 07 June 2022

The original version of this Article contained an error in the Funding section.

“This work was supported by the Natural Science Foundation of Hunan Province (S2021JJQNJJ2504).”

now reads:

“This work was supported by the Natural Science Foundation of Hunan Province (2021JJ40881).”

The original Article has been corrected.

