# Supplementary material for: Application of 5-aminolevulinic acid-mediated Waterlase-assisted photodynamic therapy in the treatment of oral leukoplakia
Source: Sci Rep. 2022 Jun 7;12:9391. doi: 10.1038/s41598-022-13497-3 (PMC9174230; doi:10.1038/s41598-022-13497-3)
Supplement: Supplementary file 1 — Supplementary Information. [file 41598_2022_13497_MOESM1_ESM.pdf]

Application of 5-aminolevulinic acid-mediated waterlaser assisted photodynamic therapy in the treatment of oral leukoplakia

Author:
Jiali, Ou[1],
Master, Department of Stomatology, The Second Xiangya Hospital, Central South University, P. R. China, consulted on literature.
139 Renmin Middle Road, Changsha 410011, P. R. China.
Tel: +86-731-85295012;
Fax numbers: +86-731-85292108
E-mail address: 198212363@csu.edu.cn

Yijun, Gao[1],
Assistant Professor, Department of Stomatology, The Second Xiangya Hospital, Central South University, P. R. China, contributed substantially to discussion.
139 Renmin Middle Road, Changsha 410011, P. R. China.
Tel: +86-731-85295012;
Fax numbers: +86-731-85292108
E-mail address: gaoyijun@csu.edu.cn.

Huan Li[2],
Master, Department of Stomatology, The Second Xiangya Hospital, Central South University, P. R. China, consulted on literature.
139 Renmin Middle Road, Changsha 410011, P. R. China.
Tel: +86-731-85295012;
Fax numbers: +86-731-85292108
E-mail address: 2701150301@ csu.edu.cn

Tianyou, Ling[3],
Professor, Department of Stomatology, The Second Xiangya Hospital, Central South University, P. R. China, proofread the manuscript.
139 Renmin Middle Road, Changsha 410011, P. R. China.
Tel: +86-731-85295012;
Fax numbers: +86-731-85292108
E-mail address: 772660166@qq.com

Xiaoyan, Xie*,
PhD Scholar, Department of Stomatology, The Second Xiangya Hospital, Central South University, P. R. China, proofread the manuscript.
139 Renmin Middle Road, Changsha 410011, P. R. China.
Tel: +86-731-85295012;

Fax numbers: +86-731-85292108
E-mail address: xyxie@csu.edu.cn

[1] These authors contributed equally to this work and should be considered first authors.
* Corresponding authors.

Acknowledgments:

This work was supported by Natural Science Foundation of Hunan Province (S2021JJQNJJ2504).

Date of submission: December 29,2021

中南大学湘雅二医院医学伦理委员会
科研项目伦理审批件

（2020）伦审第（研 520）号

<table>
<tr><td>项目名称</td><td colspan="6">光动力治疗引导血管再生在口腔黏膜下纤维性变中的作用及机制研究</td></tr>
<tr><td>研究分类</td><td colspan="3">1☐ 2☑ 3☐ 4☐</td><td>标本种类</td><td colspan="2">1☑ 2☐ 3☑ 4☐ 5☐ 6☐</td></tr>
<tr><td>申办单位</td><td colspan="6">中南大学湘雅二医院</td></tr>
<tr><td>承担专业</td><td>口腔</td><td>承担责任</td><td>负责 ☑ 参与☐</td><td>主要研究者</td><td colspan="2">谢小燕</td></tr>
<tr><td rowspan="3">提交材料</td><td colspan="2">试验方案有 ☑ 无☐</td><td rowspan="3">合作单位</td><td colspan="3" rowspan="3">无</td></tr>
<tr><td colspan="2">知情同意书有 ☑ 无☐</td></tr>
<tr><td colspan="2">参考文献有 ☑ 无☐</td></tr>
<tr><td colspan="2">专业负责人对项目涉及伦理问题的审核意见：</td><td colspan="5">该项目是☑否☐符合医学伦理原则及道德要求
专业负责人签名：[illegible] 日期：2020.9.11</td></tr>
<tr><td colspan="2">中南大学湘雅二医院医学伦理委员会意见：</td><td colspan="5">同意该项目进行申报，项目开展实施之前应向伦理委员会申请对研究方案、知情同意书等内容进行会议审查。
中南大学湘雅二医院 医学伦理委员会
2020 年 9 月 11 日
医学伦理委员会盖章</td></tr>
<tr><td colspan="7">备注：研究分类：1=观察性研究；2=试验性研究；3=理论性研究；4=其他（回顾性研究）
标本种类：1=病理组织；2=外周血；3=病例资料；4=问卷；5=细胞株；6=其他（影像等）</td></tr>
</table>
